# Targeting Motor End Plates for Delivery of Adenoviruses: An Approach to Maximize Uptake and Transduction of Spinal Cord Motor Neurons

**DOI:** 10.1038/srep33058

**Published:** 2016-09-13

**Authors:** Andrew Paul Tosolini, Renée Morris

**Affiliations:** 1 Translational Neuroscience Facility, School of Medical Sciences, The University of New South Wales, Sydney, NSW, 2052, Australia

## Abstract

Gene therapy can take advantage of the skeletal muscles/motor neurons anatomical relationship to restrict gene expression to the spinal cord ventral horn. Furthermore, recombinant adenoviruses are attractive viral-vectors as they permit spatial and temporal modulation of transgene expression. In the literature, however, several inconsistencies exist with regard to the intramuscular delivery parameters of adenoviruses. The present study is an evaluation of the optimal injection sites on skeletal muscle, time course of expression and mice’s age for maximum transgene expression in motor neurons. Targeting motor end plates yielded a 2.5-fold increase in the number of transduced motor neurons compared to injections performed away from this region. Peak adenoviral transgene expression in motor neurons was detected after seven days. Further, greater numbers of transduced motor neurons were found in juvenile (3–7 week old) mice as compared with adults (8+ weeks old). Adenoviral injections produced robust transgene expression in motor neurons and skeletal myofibres. In addition, dendrites of transduced motor neurons were shown to extend well into the white matter where the descending motor pathways are located. These results also provide evidence that intramuscular delivery of adenovirus can be a suitable gene therapy approach to treat spinal cord injury.

Recent progress in gene transfer techniques has provided the scientific community with new strategies to treat spinal cord injury (SCI). Among the different modes of gene delivery, one of the most commonly used approaches is *ex vivo* or cellular gene transfer. This technique consists of implanting, in the site of injury, cells (e.g., fibroblasts, mesenchymal stem cells, olfactory ensheathing glial cells, etc.) that have been genetically modified to express neuroprotective and/or neuroregenerative proteins such as neurotrophic factors^1^,^2^,^3^,^4^. Another common gene delivery approach consists of direct spinal cord injections of viral-vectors containing the gene sequence for a therapeutic transgene^3^. Viral-mediated transgenes can also be delivered systemically via intrathecal or intravenous routes.

Most studies aimed at repairing the spinal cord with gene therapy have used viral vectors that lead to the permanent expression of the therapeutic transgene^5^,^6^. It is worth noting that permanent expression of neurotrophic factors, such as brain-derived neurotrophic factor (BDNF), has been reported to cause spasticity and muscle hyperexcitability^7^,^8^. Transient expression of BDNF, however, encouraged the elongation of axons into a lesion cavity^9^. One of the important challenges for the treatment of SCI is to find a means to control the temporal expression of the therapeutic gene(s). In this regard, adenoviral vectors, which offer the possibility to express a therapeutic gene in a transient manner, have been used on animal models of spinal cord injury with promising outcomes^10^,^11^. Adenoviruses are non-enveloped, double-stranded DNA viruses enclosed within an 80–100 nm icosahedral-shaped protein capsid^12^. Recombinant adenoviruses (Ad) are rendered replication-deficient with the deletion of the genes responsible for viral replication (e.g., E1)^12^ and are therefore considered safe for human viral-mediated gene delivery^13^,^14^,^15^.

Another significant difficulty for gene therapy is to find a means to spatially regulate gene expression. Indeed, it is important to limit the distribution of the transgene to only one cellular component of the spinal cord as the ubiquitous expression of a therapeutic transgene could produce unwanted effects. For instance, BDNF delivery to the spinal cord protects ventral horn motor neurons (for recent review see ref. 16), but can also induce neuropathic pain in sensory neurons^17^. Viral vectors such as lentiviral vectors^18^ and adeno-associated virus (AAV)^5^,^19^,^20^ can be administered to skeletal muscle for retrograde transport along the peripheral nerve and restrict transgene expression into spinal cord or brainstem motor neurons. Ad vectors delivered intramuscularly, however, have the unique ability to restrict both the temporal and spatial expression of the transgene(s) of interest^21^,^22^,^23^,^24^,^25^.

Surveying the literature on intramuscular injections of Ad reveals important methodological differences (Table 1). These include the volumes of Ad and their viral titers, the number of days post-delivery at which the animals are euthanized and their tissues processed, the nature of the transgene of interest, the promoters that drive its expression, as well as the site where intramuscular injections are performed on the targeted muscles^21^,^22^,^23^,^24^,^25^,^26^,^27^,^28^,^29^. Overall, according to these studies, the therapeutic benefits of adenoviral-mediated intramuscular gene delivery can be described as sub-optimal. As a result, once considered a powerful alternative to direct injections or cellular gene transfer to the spinal cord, intramuscular delivery techniques to shuttle genes into spinal cord motor neurons have received less scientific attention over the last decade^30^. The aim of the present study was to systematically evaluate 1) the best injection sites in skeletal muscles for maximum transgene expression in motor neurons, 2) the time course of expression of the transgene and 3) the effect of age of the animal on transduction levels. This systematic analysis of intramuscular delivery parameters of adenovirus will improve the transfer efficacy of therapeutic genes, not only for the treatment of spinal cord injury but also for other neuromuscular dysfunctions.

## Results

### Intramuscular Injections

**Figure 1:**
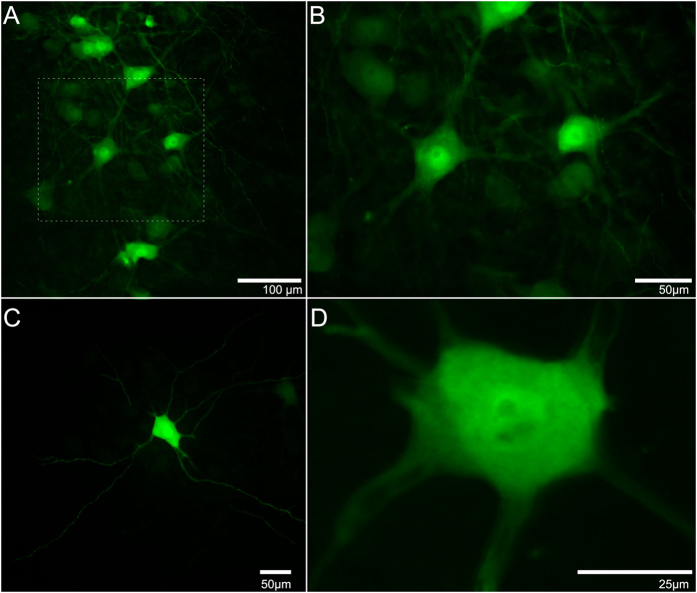
Representative images of enhanced green fluorescent protein (eGFP)-expressing motor neurons after intramuscular injections of Ad.eGFP at the motor end plate (MEP) region in triceps brachii and obtained from an adult mouse seven days after injection. (**A**) Photomicrograph of a horizontal section through the ventral horn of the 5th cervical spinal cord segment (C5) showing typical eGFP expression in motor neurons. (**B**) Magnification of (**A**). (**C**) A single motor neuron within the spinal cord to show its axon and dendritic arborisation. (**D**) A z-stack of a different motor neuron at higher magnification. Both (**C**,**D**) were obtained from an adult mouse three days after intramuscular injection.

Ad.eGFP intramuscularly delivered to the motor end plate (MEP) region of triceps brachii was internalized by the motor axon terminals and retrogradely transported to the ventral horn of the spinal cord. The transgene eGFP was translated and distributed throughout motor neuron somata and their axonal/dendritic processes. Figure 1A,B reveals the intricate network of transduced motor neurons and their afferent/efferent processes. Figure 1C,D displays the morphology of single motor neurons with eGFP expression throughout its soma and branches. In this figure, up to eight branches from a single soma expressing eGFP can be observed (Fig. 1C,D).

### Motor End Plate Targeting Analysis

**Figure 2:**
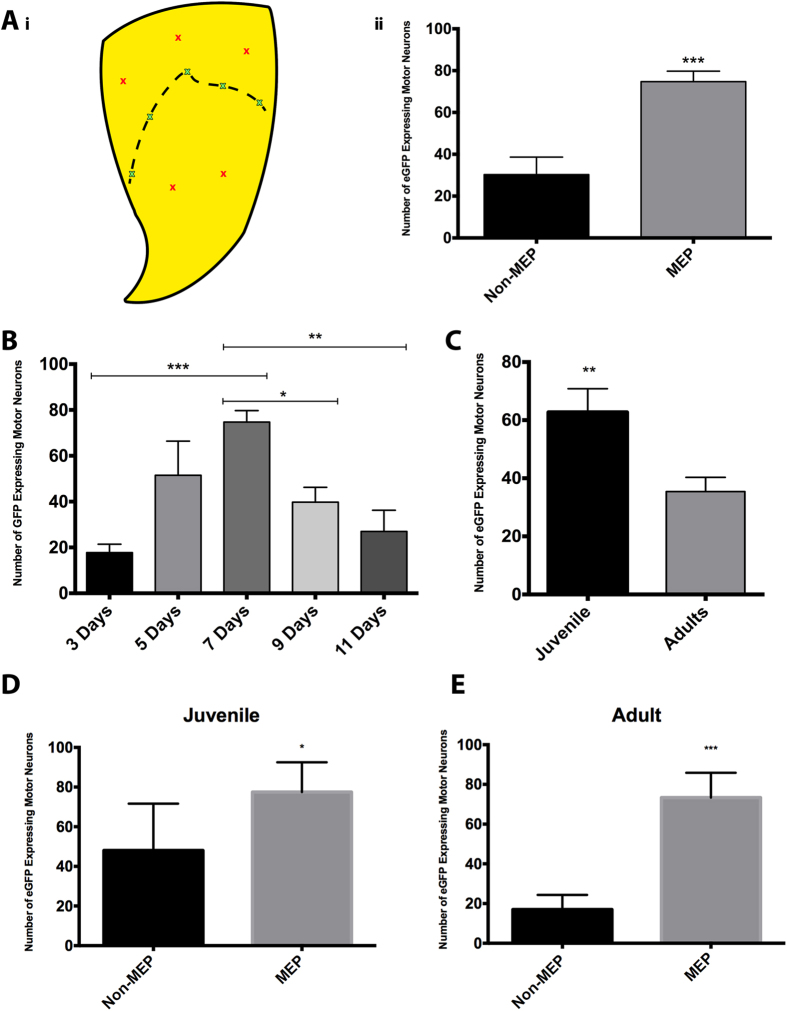
Optimisation of intramuscular delivery parameters of adenovirus. (**A**) Comparison of the efficacy of Ad.eGFP uptake between injections at and away from the motor end plate region in triceps brachii. i) Illustration of the targeted sites in triceps brachii. The black dashed line represents the location of the entire motor end plate (MEP) region as per Tosolini *et al*.^35^. The green crosses represent the five areas targeted for full-length MEP injections and the red crosses represent the areas away from the MEP region (Non-MEP). ii) Quantification of the numbers of eGFP-expressing motor neurons from both age groups. An unpaired t-test revealed a statistically significant difference between the non-MEP group and the MEP group with a two-tailed p = 0.0007 (***). (**B**) Number of Ad.eGFP-positive neurons after intramuscular injection of 40 μls of Ad.eGFP into the MEP region in triceps brachii observed after 3, 5, 7, 9 and 11 days post-injections. Unpaired t-test analyses indicated that the number of transduced motor neurons from the seven-day group was significantly larger than the three-day group (***p = 0.0002), the nine-day group (*p = 0.019) and the eleven-day group (**p = 0.0014). (**C**) Comparison of the number of transduced motor neurons after intramuscular injections of Ad.eGFP between juvenile and adult animals. T-test analysis revealed a statistical difference between the two groups (t = 3.07; df =  36; p = 0.0041). The error bars in all panels reflect the mean with SEM. (**D**) Comparison of the number of transduced motor neurons in juvenile mice after intramuscular targeting of the MEP vs non-MEP areas in triceps brachii. An unpaired t-test revealed a statistically significant difference between the age-matched groups with a one-tailed p value = 0.0485 (*). (**E**) Comparison of the number of transduced motor neurons in adult mice after intramuscular targeting of the MEP vs non-MEP areas in triceps brachii. An unpaired t-test revealed a statistically significant difference between the age-matched groups with a one-tailed p value = 0.0003 (***). The mice in which the data was obtained for (**D**,**E**) received 40 μl of Ad.eGFP distributed into the locations depicted in (**A**) and tissue was obtained seven days later.

In order to determine if targeting the MEP region with Ad.eGFP maximizes the number of transduced spinal cord motor neurons, intramuscular injections with Ad.eGFP were also performed in areas of triceps brachii that are away from the motor end plate region. Figure 2A shows that targeting the MEPs with Ad.eGFP produced a 2.5 fold increase in the number of transduced motor neurons when compared with the non-MEP group. An unpaired t-test revealed a statistically significant difference between the non-MEP group (n = 7, SEM = 8.35) and the MEP group (n = 7, SEM = 5.01) with a two-tailed p value = 0.0007 (t = 4.52, df = 12).

### Time Course of Expression

The presence of eGFP-positive motor neurons was observed in motor neurons after three, five, seven, nine and eleven days post intramuscular delivery. No eGFP expression was observed in motor neurons after fourteen, twenty-eight or forty-two days. The time course of expression for eGFP in spinal cord motor neurons exhibited a Gaussian curve-like distribution with peak expression at seven days (Fig. 2B). Statistical significance was determined between the different time points by using a one-way ANOVA (F[4, 18] = 8.98, p = 0.004). Post-hoc analysis for multiple comparisons using Bonferroni’s correction indicated that the seven-day group exhibited a greater number of transduced motor neurons than the three-day group (t = 5.25, p = 0.0002), the nine-day group (t = 3.22, p = 0.019) and the eleven-day group (t = 4.39, p = 0.0014).

### Age Matching

Data sets were divided into juvenile and adult groups to see if the age of the mice at the time of surgery could influence the number of eGFP-expressing motor neurons. Animals aged between 5–7 weeks (n = 14) at the time of Ad.eGFP intramuscular injections were assigned into the juvenile group and can be further categorised into the following age brackets: 5 weeks old (n = 4), 6 weeks old (n = 2) and 7 weeks old (n = 8). The adult cohort were those aged 8 weeks and above (n = 24) and can be further categorised into the following age brackets: 8 weeks old (n = 4), 10 weeks old (n = 2), 12 weeks old (n = 4), 13 weeks old (n = 2), 15 weeks old (n = 4), 16 weeks old (n = 4) and 20 weeks old (n = 4). Unpaired t-test analysis revealed that the numbers of Ad.eGFP transduced motor neurons were significantly greater in the juvenile group when compared with the adult group (t = 3.07; df = 36, p = 0.0041) (Fig. 2C). In addition, comparisons between the numbers of transduced motor neurons after targeting the triceps MEP vs non-MEP regions were performed individually, for both juvenile and adult mice. For the juvenile mice, an unpaired t-test revealed that Ad.eGFP transduced more motor neurons when targeting the MEPs (n = 4) than targeting non-MEP (n = 3) regions (t = 2.040, df = 5, p = 0.0485) (Fig. 2D). For the adult mice, an unpaired t-test revealed more transduced motor neurons when targeting the MEPs (n = 3) than targeting non-MEP (n = 4) regions (t = 7.570, df = 5, p = 0.003) (Fig. 2E).

### Cocktail Injections of Ad.eGFP and Tetramethylrhodamine (Mini-Ruby)

**Figure 3:**
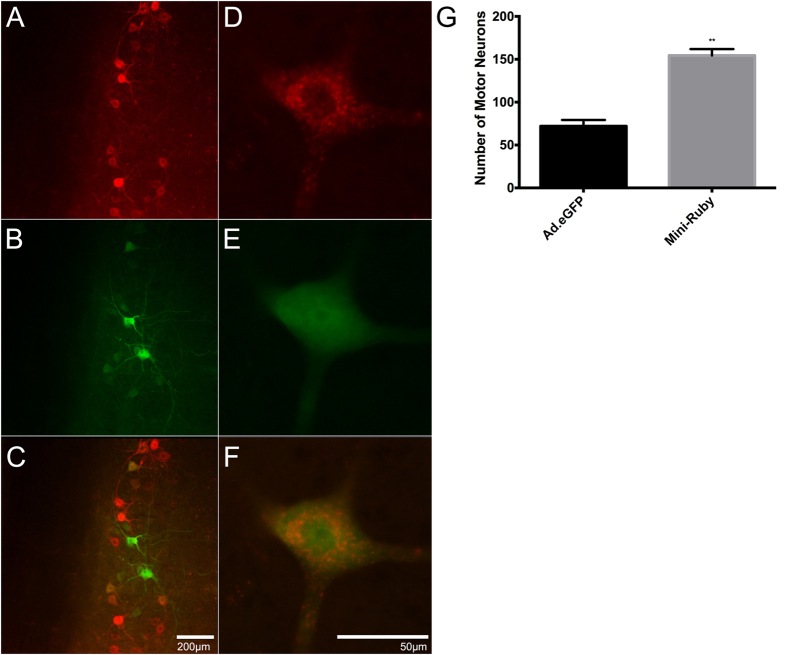
Representative images of Ad.eGFP and Mini-Ruby-positive motor neurons in the 7th cervical spinal cord segment (C7), seven days after 40 μl intramuscular injections of the cocktail at the MEP region in triceps brachii. The TRITC and the FITC channels allow for the visualisation of Mini-Ruby (**A**) and eGFP (**B**), respectively. (**C**) Overlay of the two channels. Scale bar = 100 μm. (**D**–**F**) Higher magnification of a double-positive eGFP expressing and Mini-Ruby labelled motor neuron from a different section to that found in (**A**–**C**). Scale bar = 50 μm. (**G**) Quantitative analysis comparing the number of Ad.eGFP-transduced and Mini-Ruby-labelled motor neurons after intramuscular injections of a cocktail containing both constituents. T-test analysis reveals a statistical difference between the two groups (t = 7.89; df = 4; p = 0.0014). The error bars reflect the mean with SEM. Note, all cocktail-injected data were obtained from mice seven weeks of age and tissue was obtained seven days later.

Cocktail injections of Ad.eGFP and Mini-Ruby in triceps brachii resulted in a combination of single eGFP-expressing, single Mini-Ruby-labelled as well as eGFP-expressing/Mini-Ruby–labelled motor neurons (Fig. 3). Columns of eGFP-expressing- or Mini-Ruby-labelled motor neurons were found to span a similar number of spinal cord segments compared to those obtained after Ad.eGFP or Mini-Ruby injections alone (i.e., C5-T1). Moreover, the number of transduced motor neurons from the cocktail injections was similar to that of Ad.eGFP only injections. These data indicate that intramuscular injections of Ad.eGFP and Mini-Ruby does not appear to affect the expression or labelling patterns of each of the individual constituents of this cocktail. Qualitative analysis showed that there were more Mini-Ruby labelled motor neurons than eGFP-expressing motor neurons (Fig. 3A–C). Figure 3D–F illustrates the typical punctate appearance of Mini-Ruby distributed throughout the cytoplasm and dendritic processes of the motor neurons, a labelling profile that is in sharp contrast with the smooth ubiquitous appearance of eGFP expression. Statistical analysis revealed that there were significantly more Mini-Ruby labelled motor neurons than eGFP-expressing motor neurons (t = 7.89, df = 4, p = 0.0014) (Fig. 3G).

### Motor Neuron Axonal and Dendritic Pattern of eGFP Expression

**Figure 4:**
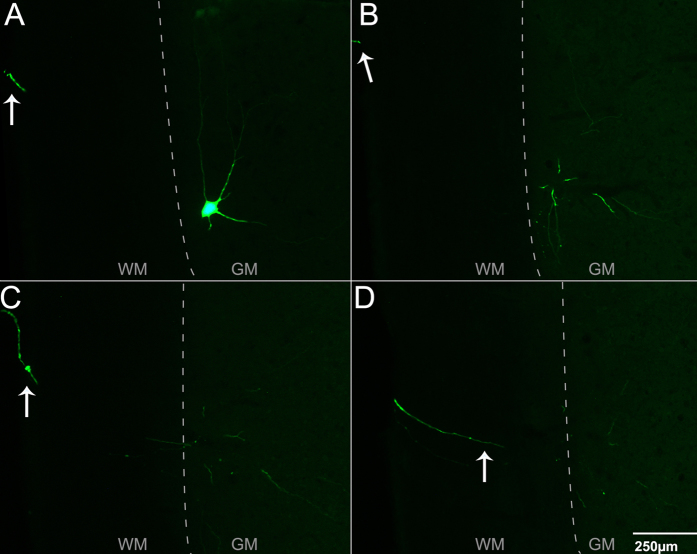
Representative images of a series of horizontal sections through the ventral horn of the 7th cervical spinal cord segment (C7) three days after intramuscular injections of Ad.eGFP at the motor end plate region in triceps brachii were performed. (**A**–**D**) Consecutive 50 μm-thick ventral-to-dorsal series of images showing the axonal/dendritic processes from one single transduced motor neuron. The arrows indicate the presence of a single process projecting from the soma (in A) through the ventral white matter. Numerous dendrites arising from the motor neuron can also be observed throughout the local spinal cord. These images were obtained from an adult mouse three days after injections. GM: grey matter; WM: white matter. Scale bar = 250 μm.

**Figure 5:**
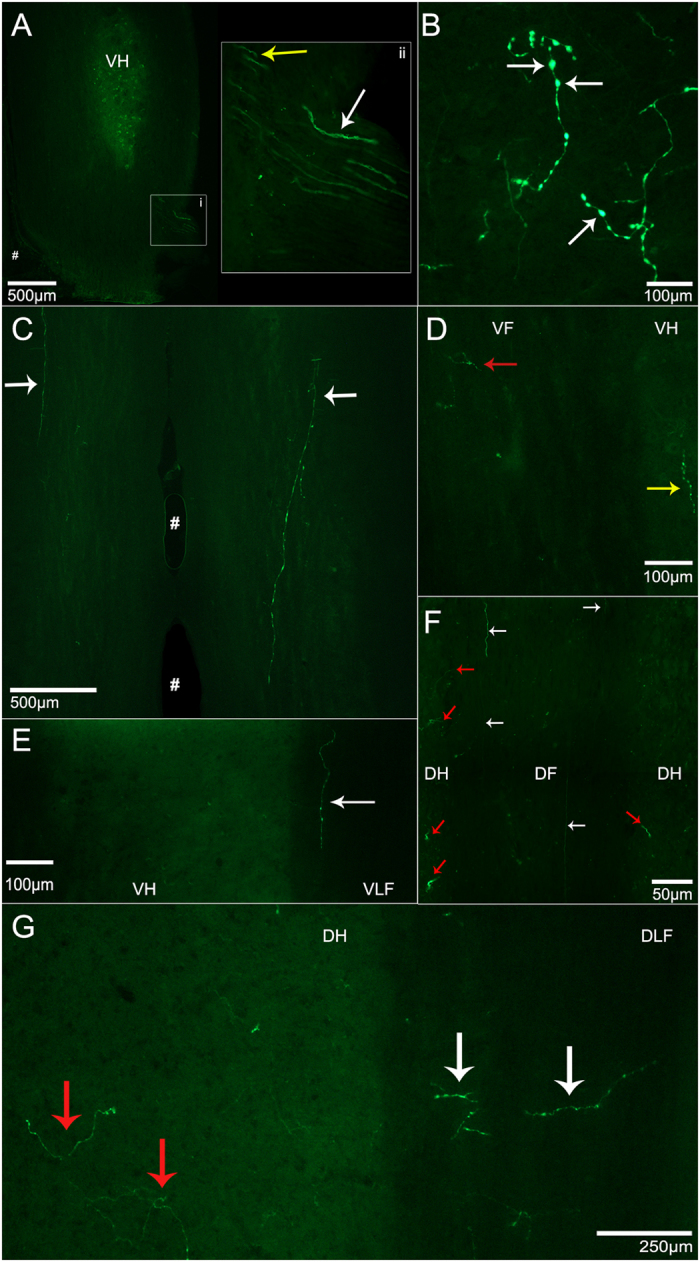
Representative images of eGFP-expressing axonal and dendritic processes extending from transduced motor neurons after Ad.eGFP injections were performed into the motor end plates of triceps brachii. (**A**) Longitudinal sections through the ventral horn (VH) of the cervical spinal cord showing eGFP-expressing axons from triceps brachii transduced motor neurons extending through the ventrolateral funiculus (VLF) to exit via ventral roots. *i*) Axons of transduced motor neurons extending into the ventral root. *ii*) A close-up of *i*). White arrows indicate axons expressing eGFP extending into the ventral roots whereas the yellow arrows indicate the eGFP-expressing axons extending through the white matter located in the VLF. (**B**) eGFP-expression in processes located locally in VH grey matter. White arrows are suggestive of axonal boutons. (**C**) Longitudinal section of the white matter ventral to the VH in the cervical spinal cord after bilateral Ad.eGFP. White arrows indicate eGFP-expressing dendritic processes running through two cervical segments of the VLF. (**D**) Cervical section showing the VH and the ventral funiculus (VF). Red arrow indicates eGFP-expressing fibres located within the VF, whereas the yellow arrow indicates eGFP-expression located in the medial aspect of the VH. (**E**) Longitudinal section that includes the right VH and VLF. The white arrow points to one eGFP-expressing process located in the VLF. (**F**) Longitudinal section through the cervical spinal cord showing eGFP-expression in processes extending into the dorsal funiculus (DF). eGFP-expressing processes are extending along the rostro-caudal axis through the DF as indicated by white arrows and through the dorsal horn grey matter into the DF as indicated by red arrows. (**G**) eGFP-expressing processes extending into the DH and dorsolateral funiculus (DLF). White arrows indicate eGFP-expressing fibres extending into the DLF whereas the red arrows indicate eGFP-expressing processes extending into the DH. Images were obtained from mice from a variety of timepoints ranging from days 3–11. VH: ventral horn, VLF: ventrolateral funiculus, VF: ventral funiculus, DH: dorsal horn, DF: dorsal funiculus, #: the ventral median fissure.

**Figure 6:**
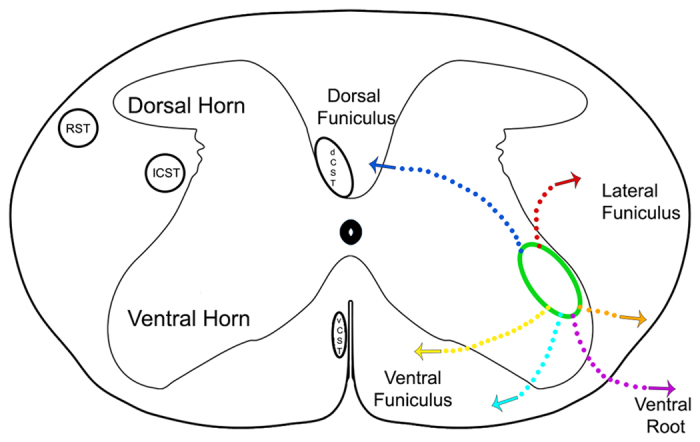
Diagrammatic representation of the location of eGFP-expressing axonal/dendritic processes that extend into different compartments of the white matter where the main descending motor tracts are located. The green oval represents the pool of motor neurons that supply triceps brachii as per Tosolini *et al*.^34^. Each coloured line represents the location of eGFP-expressing processes in the white matter as observed in Fig. 5. RST: rubrospinal tract; dCST: dorsal corticospinal tract; lCST: lateral corticospinal tract; vCST: ventral corticospinal tract.

**Figure 7:**
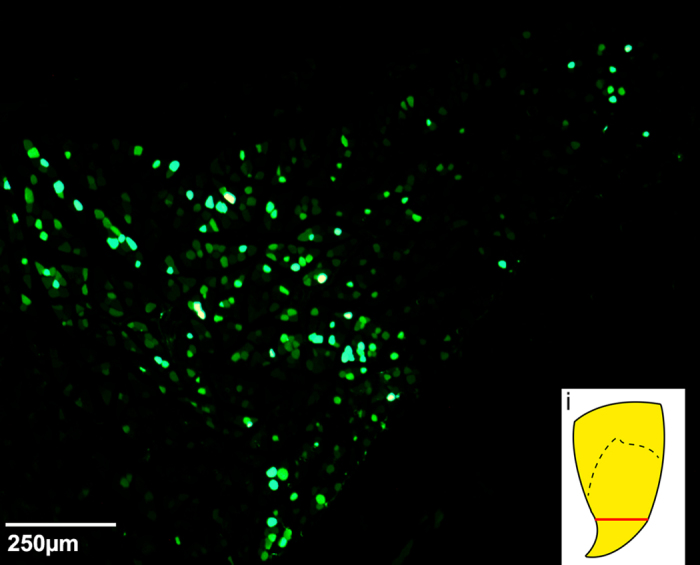
Representative transverse section through the triceps brachii muscle showing eGFP expression in the muscle fibres. This muscle tissue was collected one week after intramuscular injections were performed into this muscle. (**A**) This section is located at the distal end of the muscle in an area that is distal to the motor end plate region, as indicated by the red line in *i*).

Targeting the motor end plate region of triceps brachii with Ad.eGFP resulted in widespread expression of the transgene. Figure 4 is a series of four consecutive 50 μm-thick sections through the ventral horn of the spinal cord displaying axonal and dendritic branching from one transduced motor neuron. Figure 5A shows a longitudinal section through the ventral horn of the spinal cord with eGFP-expressing motor axons exiting the ventral horn via the ventrolateral funiculus and entering the ventral roots. Locally in the ventral horn grey matter, eGFP-expressing dendritic processes with numerous possible spines were observed (Fig. 5B). Figure 5C shows eGFP-expression in processes also located in the ventrolateral funiculus after bilateral Ad.eGFP injections into triceps brachii. In this section of tissue, eGFP-expressing processes extend, at least, through two spinal cord segments. Furthermore, eGFP-expression was also found in processes extending medially into the ventral funiculus (Fig. 5D) as well as laterally (Fig. 5E) into the ventrolateral funiculus as well as, in the dorsal funiculus (Fig. 5F) and dorsolateral funiculus (Fig. 5G). Figure 6 summarizes the course taken by the eGFP-transduced axonal and dendritic processes extending from the grey matter toward and into the different motor tracts running within the white matter of the spinal cord. In addition to eGFP-expression found in the spinal cord, Ad.eGFP-targeted muscles exhibited robust expression of eGFP in the majority of muscle fibres (Fig. 7). These eGFP-expressing muscle fibres were located both proximal and distal to the injection sites (as shown in Fig. 7i).

## Discussion

The present study is a systematic evaluation of the parameters for the maximum transgene expression in spinal cord motor neurons after intramuscular delivery. These parameters are 1) the location of the injection sites within skeletal muscles, 2) the time course of expression of the transgene in spinal cord motor neurons and 3) the influence of the age of the animals on motor neuron transduction levels. Targeting the motor end plates (MEPs) in triceps brachii with Ad.eGFP yielded a 2.5-fold increase in the number of transduced motor neurons when compared with the delivery of the same volumes of adenoviral vectors in regions of the muscle that are away from the MEP region. Peak expression of the transgene was detected seven days after the injections were performed. In addition, more motor neurons were transduced in juvenile (i.e., 5–7 weeks old) than in adult mice (i.e., 8+ weeks old). In addition, eGFP-expressing dendrites from transduced motor neurons were observed to extend well into white matter regions where the different descending motor pathways run. This analysis also showed that targeting the MEPs with a cocktail of Ad.eGFP and the retrograde tracer Mini-Ruby was a practical option to spatially determine the location of transduced motor neurons after adenoviral-mediated transgene expression had faded.

### Targeting the Motor End Plate Region for Maximum Transgene Expression

Once considered a promising technique to restrict transgene expression into ventral horn motor neurons, the popularity of intramuscular injections of adenoviral vectors have diminished over the last decade, mainly because it has resulted in suboptimal levels of transduction. In the literature, the exact location of the intramuscular injections of recombinant adenoviral vectors is not always disclosed in great detail, however most intramuscular injections of adenoviral vectors seem to be performed in the ‘belly’ of the muscle^25^,^29^,^31^,^32^,^33^ (Table 1). Over the last years, we have mapped the location of the motor end plates of the mouse and the rat forelimb and hindlimb and found that to maximise the delivery of retrograde tracers, the entire MEP region needs to be targeted^34^,^35^,^36^,^37^,^38^. Here, we demonstrated that this principle also applies to recombinant adenoviral vectors. Indeed, the present analysis showed that targeting the entire MEP region of triceps brachii with Ad.eGFP lead to approximately a 2.5-fold increase in the number of transduced motor neurons in comparison with injections that were directed to areas of the muscles distal to the MEPs. This was also the case for when the analysis was performed separately, both juvenile and adult mice whereby targeting the MEPs resulted in significantly more transduced motor neurons. Hence, regardless of age it is clear that targeting the MEPs results in greater transduction efficiency of spinal cord motor neurons.

Adenoviruses enter cells through receptor-mediated endocytosis where they bind with high affinity to receptors such as coxsackie and adenovirus receptors (CAR) and members of the integrin receptor family, amongst others^39^,^40^,^41^. In skeletal muscle, CARs are confined to the neuromuscular junction in adult animals^40^,^42^ and after internalization, adenoviral particles are sorted and trafficked by cytoplasmic dynein along the microtubule network to the cell soma where they gain entry into the nucleus to induce gene expression^39^,^41^. This, at least partially, explains the success of the MEP targeting. The present study, therefore, suggests that the sub-optimal levels of motor neuron transduction reported in the literature after intramuscular injections of adenoviral vectors may not only be due to the choice of virus, the expression cassette or the gene of interest but, also, to poor uptake at the neuromuscular junction.

### Time Course of Expression of Ad.eGFP

In line with the literature, the present investigation showed that expression of the eGFP transgene could be observed in motor neurons as soon as three days post delivery and up to eleven days, with expression levels peaking at seven days^21^,^22^,^29^,^31^,^32^,^43^,^44^,^45^. These data represent both age groups, as age-dependent time-course of expression experiments were not performed. There have been reports, however, that transgene expression after intramuscular injections can last for much longer (i.e., up to four months), specifically in new-born animals^23^,^27^,^46^ or when measured by RT-PCR techniques^27^. This temporal regulation of adenoviral-mediated gene expression is advantageous where short-term up-regulation of the transgene is advantageous. In particular, transient gene expression can be beneficial for spinal cord regeneration whereby tightly regulated control of synaptogenesis is required between descending motor axons and their targets, motor neurons and interneurons^6^,^47^,^48^,^49^. In this context, short-term neurotrophic factor treatment facilitates synaptic plasticity^9^,^16^ whereas chronic treatment has resulted in spasticity and hyper-excitability^7^,^8^, due to the probable formation of novel, aberrant connections^6^,^7^,^8^,^30^. Further, short-term expression of neurotrophic factors fits the growth rate of regenerating axons, which is estimated to be 1 mm per day^50^. Therefore, the auto-regulatory temporal gene-expression, as mediated by the transient nature of adenovirus, is ideal for short-term expression of therapeutic genes.

### Age Matching

In our hands, the total numbers of transduced motor neurons arising from intramuscular injections of Ad.eGFP was greater in juvenile mice. A caveat, however, is that this statistical analysis was conducted post-hoc on all data sets, (i.e., time-course and optimal injection sites). Despite this limitation, we are confident that the success of intramuscular delivery and retrograde transport of adenovirus is age-dependent. One explanation for the greater numbers of transduced motor neurons observed in the juvenile group could be, at least in part, attributable to the fact that they received the same volume of Ad.eGFP as the adult cohort, despite that they would have smaller triceps. We do not think, however, that the difference in muscle mass between the juvenile and the adult cohorts is the main factor that contributes to the difference in the numbers of transduced motor neurons between the two groups of mice. Rather, coxsackie and adenovirus receptor (CAR) expression would be the key factor accounting for this disparity. Indeed, other groups of researchers have established that the age of the animals directly influences vector-spread, cellular tropism and, ultimately, transgene expression^51^,^52^. It has also been established that the abundance of myoblasts and myotubes as well as the relative abundance of CARs found in young mice contributes to greater transduction efficiency^42^,^53^,^54^. Further, the overexpression of CARs in muscle from adult transgenic mice has been shown to produce a significant increase in the transduction of skeletal muscle^55^,^56^. Taken together, these findings support the view that the reduced numbers of transduced motor neurons in adult mice is more than likely a consequence of the down-regulation of CARs observed throughout age.

These data demonstrate that the retrograde machinery of motor neurons for adenoviral transport is functional from 5 weeks through to 20 weeks of age, in which period of time the ability for uptake from the MEPs into pre-synaptic axon terminals and subsequent delivery is preserved. Indeed, for the wild-type mouse, the formation of the neuromuscular junction begins during embryonic development (for a recent review see ref. 57), however, the pre-synaptic axonal terminals are in an adult-like state after one month^58^. Furthermore, as there are no differences in the signalling endosome dynamics of retrograde axonal transport of wild-type mice aged from 1 month up to 13 months old^59^ the motor neuron transduction differences cannot be due to delays in retrograde axonal transport. In summary, although it has been previously reported that gene transfer is greater in younger animals, the present study is the first to report that adenoviral delivery in skeletal muscles leads to significantly greater levels of transduction in spinal cord motor neurons of juvenile mice.

### Putative Mechanism(s) Underlying Adenoviral Vectors Transient Expression

The most common explanation for the transient expression of adenovirus is the silencing of the episomally located expression cassette, although some authors have suggested that this phenomenon is caused by an immune response triggered toward the virus from the host tissue. For instance, Zirger *et al*.^60^ have demonstrated that direct brain injections result in CD4+ and CD8+ T cell-mediated elimination of adenoviral transgene expression through both cytotoxic and non-cytotoxic mechanisms^61^. On the other hand, intramuscular injections of adenoviral particles in the diaphragm has been reported to initiate an immune response in the targeted muscle, however the central nervous system (CNS) was not scrutinized in this study to see whether the immune response has spread centrally^62^. In another study, no significant loss of motor neurons was reported after peripheral injections of adenoviral-vector in the sciatic nerve^63^, supporting the notion that the immune system is activated after central but not after peripheral nerve or muscle injections.

### Cocktail Injections of Adenoviral Vectors and Retrograde Neuronal Tracers

As adenoviral expression is transient, intramuscular injections of a cocktail of adenovirus and permanent neuronal retrograde tracer enables to decipher the exact rostro-caudal location, within the spinal cord, of transduced motor neurons after the peak of the protein marker has faded^28^. In our hands, cocktail injections lead to approximately twice as many Mini-Ruby-labelled motor neurons than eGFP-expressing motor neurons. In line with Martinov *et al*.^28^, these cocktail injections resulted in both single Mini-Ruby-labelled and eGFP-expressing motor neurons as well as double-labelled motor neurons. It is worth noting that, unlike that of adenovirus, the mechanism of uptake of dextran conjugates such as Mini-Ruby is not receptor-mediated endocytosis, perhaps explaining why the tracer and the adenoviral vector are not always co-localized inside the same motor neurons. Despite a difference in their expression patterns, both Ad.eGFP- and/or Mini-Ruby-positive motor neurons span the same number of spinal cord segments and as such, cocktail injections are a viable approach to determine the spatial distribution of transduced motor neurons after transgene expression has faded.

### Axonal and Dendritic Patterns of eGFP Expression

After the retrograde transport of recombinant Ad.eGFP, translated eGFP does not require active transport and freely diffuses throughout the transduced neurons^64^. As expected, eGFP expression was ubiquitously distributed throughout the cytoplasm, nucleus and axonal and dendritic processes of the transduced motor neurons, giving them a Golgi stain-like appearance. Interestingly, eGFP-positive dendrites were observed to extend into several compartment of the white matter (as summarized in Fig. 6). The eGFP-positive processes extending in the ventral roots are presumably axons originating from transduced motor neurons and travel within the peripheral nerve toward the targeted muscles (Fig. 5A). On the other hand, dendrites expressing eGFP were observed extending away from the neuron somata to reach into various regions of the spinal cord white matter, i.e., in close proximity to the major descending motor tracts (Fig. 5B–G). For instance, eGFP-positive dendrites were observed to extend into the dorsal funiculus where the dorsal corticospinal tract is located, the dorsolateral funiculus where both the rubrospinal and the lateral corticospinal tracts travel as well as into the ventral funiculus where the ventral corticospinal tract and the reticulospinal tracts run^65^,^66^. The extensive dendritic arborisation pattern of motor neurons suggests that many synaptic contacts between the main descending motor tracts and spinal cord motor neurons would be axo-dendritic in nature. Together these data show that motor neuron dendrites can extend a relatively great distance away from their somata to reach for synaptic contact^67^,^68^.

### Implications of the Current Work and Future Direction

This work provides a consistent adenoviral-mediated gene delivery approach for gene therapy scenarios that aim to overcome the deleterious effects of SCI and other neuromuscular dysfunction. Intramuscular injections of viral vectors are a minimally invasive and therefore constitute a clinically relevant way to deliver therapeutic genes to motor neurons^29^. Intramuscular injections of recombinant adenovirus transduce both the skeletal muscle fibres and the motor neurons that supply them, thus delivering therapeutic genes to both the central and peripheral aspects of the motor units is of interest for amyotrophic lateral sclerosis.

One question remains to be answered: is a 2.5-fold increase in therapeutic genes sufficient to promote CNS plasticity and functional recovery? A number of studies have shown that the introduction of neurotrophic factor in the injured spinal cord encourages the elongation of injured axons toward the exogenous neurotrophic source^3^,^4^,^5^,^6^,^9^,^16^,^49^. As transduced dendrites are not confined within the limits of the grey matter but extend well into the different regions of the white matter, (i.e., where descending motor tracts run) has great relevance for spinal cord regeneration. For example, in a gene therapy scenario, transduced motor neurons expressing exogenous chemoattractant molecules would become ‘therapeutic bait’ for the transected axons and that the latter will not have to leave the white matter in order to make axo-dendritic contact with the motor neurons that were once their post-synaptic targets (see 6, 50). These considerations do not determine whether intramuscular delivery of adenoviral vectors is sufficient to trigger functional regeneration. The current study, however, by improving the delivery parameters to achieve up-regulation of gene expression in spinal cord motor will contribute to making gene therapy a step closer to treat SCI and other neuromuscular disorders.

## Methods

### Animals

All experimental protocols were approved by the Animal Care and Ethics Committee of the University of New South Wales, Australia and were performed in accordance with the National Health and Medical Research Council of Australia regulations for animal experimentation. A total of 49 adult male C57BL/6 mice (ARC, Western Australia) weighing between 18 and 38 g at the time of surgery were used in this study. The mice were housed in groups of five in an animal holding room under 12-h light–dark cycle. Water and chow were freely available throughout the course of the experiments.

### Adenovirus

An expression cassette comprising the cytomegalovirus promoter and the cDNA encoding enhanced green fluorescent protein (eGFP) packaged into Ad serotype 5 was obtained from the University of Pennsylvania’s Penn Vector Core (Ad.eGFP). There were two separate batches of adenovirus used in this study. The viral titre of the first batch was 4.84 × 10^12^ pfu/ml whereas the viral titre of the second batch was 5.48 × 10^12^ pfu/ml as quantified using real-time PCR primer-probe sets specific for the E2a gene (data not shown). Each experimental group received intramuscular injections from both adenoviral batches. For example, for the seven-day experimental group, four animals received adenovirus from the first batch (i.e., 4.84 × 10^12^ pfu/ml) and three animals received adenovirus from the second batch (i.e., 5.48 × 10^12^ pfu/ml).

### Intramuscular Injections in Triceps Brachii

Anesthesia was induced with isoflurane (Provet, Sydney, NSW, Australia; 1–2% in O_2_). The fur covering the targeted forelimb was shaved and cleaned with 70% ethanol. A small incision was made directly in the skin to expose triceps brachii. Each animal received 40 μl of Ad.eGFP delivered in five injections evenly distributed along the full length of the motor end plates (MEPs) with graded glass micropipettes with lumen size at the tip of the micropipettes of 0.5 μm (DKSH, Zurich, Switzerland) as per Mohan *et al*.^37^. These 1 μl-graded glass micropipettes have a maximum volume capacity of 10 μl. The Ad.eGFP-filled micropipettes were slowly lowered in the muscle until a slight resistance is encountered that indicates that the tip of the micropipettes has reached the fasciae that cover the deepest part of the muscle. The adenovirus is then slowly delivered manually, 1 μm at a time, while the tip of the micropipette is steadily lifted towards the exposed surface of the muscle. The motor end plate region for triceps brachii forms an upside down ‘V’ shape and traverses the entire width of the muscle^35^. Great care was taken to preserve the fasciae covering the muscle and those in the surrounding as well as to spare the blood vessels near the targeted area. After the injections, the muscle was wiped with gauze to remove any virus that may have inadvertently seeped out from the injected muscle, after which the skin was closed with surgical clips (Texas Scientific Instruments LLC, Boerne, TX, USA). In order to determine whether targeting the motor end plates would lead to maximum uptake of adenovirus and expression of the transgene, 40 μl of Ad.eGFP was also injected in areas of triceps brachii that surround, but do not include, the MEP region (n = 7).

### Time Course of Expression of Ad.eGFP

In order to establish the time course of expression of the eGFP reporter gene, 40 μl of Ad.eGFP was injected along the entire motor end plate region of triceps brachii. The mice were then killed at 3 (n = 4), 5 (n = 4), 7 (n = 7), 9 (n = 4), 11 (n = 4), 14 (n = 10), 28 (n = 4) and 42 (n = 6) days post-intramuscular injections.

### Cocktail Injections of Ad.eGFP and Mini-Ruby

Cocktails containing 40 μl of Ad.eGFP and 6 μl of the biotinylated dextran tetramethylrhodamine Mini-Ruby (10 000 MW; 5% in distilled water; Life Technologies, Mulgrave, VIC, Australia) were delivered in triceps brachii. All cocktail-injected mice were 7 weeks of age (i.e., juvenile) at the time of injections and were perfused 7 days later (n = 3).

### Tissue Collection and Histological Processing

At the conclusion of these experiments, the mice received a lethal dose of Lethabarb (100 mg/ml; Virbac, Sydney, NSW, Australia) and were intracardially perfused with 0.1 M phosphate buffer (PB) followed by 4% paraformaldehyde (Sigma Aldrich, Castle Hill, NSW, Australia) in 0.1 M PB. Cervical spinal cord dissections were performed by making an incision into the skin covering the midline of the animal, from the base of the skull to the thoracic region (as per 35, 37). The paravertebral muscles were then reflected, exposing the cervical part of the vertebral column and the large spinous process of C2 was identified and then removed. This process exposed the dorsal roots of C2, which were then colored with a permanent marker for easy identification. C3–T1 vertebrae were subsequently removed, one at a time, exposing their dorsal roots that were also coloured with permanent markers using alternative colours (e.g., C3, C5, and C7 were coloured with a green marker and C4, C6 and C8 were coloured with a blue marker). A fiducial mark was made in the white matter approximately half way between two adjacent dorsal roots to indicate segmental boundaries. After this process, the cervical spinal cord was cut transversely into two-segment blocks (i.e., C3-C4, C5-C6, C7-C8 blocks). The spinal cord tissue blocks were then removed from the carcass, post-fixed overnight in a solution containing 4% paraformaldehyde in 0.1 M PB and then cryoprotected in a 30% sucrose solution (Sigma-Aldrich, Castle Hill, NSW Australia) in distilled water for 2 days at 4 °C. The spinal cord blocks were then cut along the horizontal axis into 50 μm-thick sections using a cryostat. The tissue sections were collected into 48 well plates containing 0.1 M PB and then mounted onto Superfrost^TM^ Plus microscope slides (Thermo Scientific, Scoresby, VIC, Australia). Triceps brachii muscles were also dissected, post-fixed, cryoprotected and sectioned at 50 μm. The muscles were oriented so to obtain tissue sections that are transverse to the myofibres direction. The tissue was mounted directly onto Superfrost^TM^ Plus microscope slides (Thermo Scientific, Scoresby, VIC, Australia). The microscope slides were air-dried and then coverslipped with a mounting medium suitable for fluorescence microscopy (Dako, North Sydney, NSW, Australia).

### Image Analyses

An epifluorescence BX51 (Olympus, Notting Hill, VIC, Australia) and a Zeiss Z1 AxioExaminer NLO710 confocal microscopes (Carl Zeiss Pty Ltd, North Ryde, NSW, Australia) were used to capture images of the spinal cord and muscle tissue. All images were processed with Adobe Photoshop CS6 (Adobe Systems Incorporated, San Jose, CA, USA). Motor neurons were considered positively labelled when eGFP expression and/or Mini-Ruby labelling were present within both the soma and at least one axonal/dendritic process. The total numbers of eGFP- or Mini Ruby-labelled motor neurons were then counted.

### Statistical analysis

Comparisons between two groups of animals were analysed using unpaired, two-tailed t-tests. Comparisons between several groups of animals (e.g., time course of expression) were performed using a one-way analysis of variance (ANOVA) followed by *post hoc* Bonferroni’s correction. Statistical significance was determined using Prism version 6 (GraphPad Software, La Jolla, CA, USA).

## Additional Information

**How to cite this article**: Tosolini, A. P. and Morris, R. Targeting Motor End Plates for Delivery of Adenoviruses: An Approach to Maximize Uptake and Transduction of Spinal Cord Motor Neurons. *Sci. Rep.* **6**, 33058; doi: 10.1038/srep33058 (2016).

## Figures and Tables

**Table 1:** A summary of the previous literature focusing on adenoviral-mediated gene delivery to motor neurons via intramuscular injections.

Authors	Volume and Concentration of Adenovirus	Species/Strain	Age at Injection	Post-operational Time	Promoter	Gene of Interest	Injection Site	Transduction Site
Ghadge *et al*.^21^	5–70 μl of 0.5–1.0 × 10^10^ pfu/ml,	SJL/J Mice	4–6 weeks old	1–10 days	CAG	LacZ	Tibialis Ant., Tongue	Lumbar ventral horn, Hypoglossal Nucleus
Petrof *et al*.^62^	200 μl of 3×10^12^ pfu/ml	MDX and C57Bl6 mice	5–8 weeks old	1 week	RSV, CMV	Luc, LacZ	2 or 3 separate injections in the hemidiaphragm	Only muscle was analysed
Giménez y Ribotta *et al*.^69^	4 μl of 1.2–4 × 10^7^ pfu/ml	SD Rats	Post natal day 1	7 days	RSV	LacZ, BDNF, GDNF	Nasolabial or lower lip muscles	Facial Nuclei
Haase *et al*.^23^	100 μl of 1 × 10^9^ pfu/ml	PMN Mice	3–5 days old	Up to 88 days	RSV	Luc, NT-3	Gastrocnemius, Triceps Brachii, Dorsal Trunk muscles	Muscles and L2-L4 Spinal Cord Segments
Baumgartner and Shine^22^	10 μl of 1.9 × 10^8^ pfu/ml	SD Rats	Newborn	7 days	RSV	β-Gal, BDNF, CNTF, GDNF	Facial muscles of the cheek, lower lip and whisker pad	Facial nucleus
Baumgartner and Shine^26^	10 μl of 1.9 × 10^8^ pfu/ml 10 μl of 5 × 10^8 ^pfu/ml	SD Rats	1 day old	7 days, 21 days or 42 days	RSV	NT-3, CNTF, BDNF, GDNF, β-Gal,	Three different sites in the Gastrocnemius, Flexor Longus Digitorum and Tibialis Anterior muscles	Lumbar ventral horn
Baumgartner and Shine^27^	15 μl of 3 × 10^8 ^pfu/ml	SD Rats	1 day old	At 2 days facial nerve was severed. 4 or 20 weeks	RSV	GDNF, β-Gal,	Nasiolabialis, Frontalis and Auricularis Anterior muscles	Facial nucleus
Haase *et al*.^74^	1 × 10^9 ^pfu/ml	PMN Mice	3–5 days old	40–88 days	RSV	NT-3	Gastrocnemius, Triceps Brachii, Dorsal Trunk muscles	Lumbar ventral horn
Glatzel *et al*.^24^	3 μl of 2.5 × 10^7 ^pfu/ml	C57BL/6 Mice	Unknown	7–21 Days	CMV, RSV	LacZ	Tibialis Cranialis	Not observed
Soudais *et al*.^70^	3–5 × 10^12 ^pfu	Swiss OF1 Mice	4 days old	24 Days	CMV	GFP	Gastrocnemius	Sacral Dorsolumbar Rachis Ventral Horn
Yamashita *et al*.^45^	5–10 μl of 0.5–1 × 10^11 ^pfu/ml	SOD1-G93A and B6SJL Mice	1–35 weeks old	2–5 days, 1, 2 and 4 weeks	CAG	LacZ, Bcl-2, Cre	Middle or right side of the tongue	Hypoglossal Nucleus
Acsadi *et al*.^46^	5 μl of 5 × 10^9 ^pfu/ml	SOD1-G93A Mice	5–7 days old	2, 3 and 4 months	CMV	GDNF, eGFP, LacZ	Tibialis anterior, Gastrocnemius, Quadriceps and Paraspinal muscles	Lumbar ventral horn
Martinov *et al*.^28^	2–8 μl of 1.0 × 10^12 ^pfu/ml	NMRI Mice, Wistar Rats	Adult	6–41 days	CMV	eGFP, Luc	Soleus, extensor digitorum longus	Lumbar ventral horn
Millecamps *et al*.^71^	10^8 ^pfu/μl	C57BL/6, SOD1-G93A mice	45 or 130 days old	8 days	PGK, RSV	Luc, β-Gal,	4 sites in the tongue, 2 sites in Triceps Brachii and 2 sites in Gastrocnemius	Hypoglossal Nucleus
Yamashita *et al*.^72^	5–10 μl of 0.3×10^11 ^pfu/ml	SOD1-G93A and B6SJL mice	10 weeks	25 weeks	CAG	Bcl-2, Cre	Middle or right side of the tongue	Hypoglossal Nucleus
Tsai *et al*.^73^	10 μl of 6.0 × 10^8 ^pfu/ml,	FVB/NJ mice	12 day old	4–9 days	PGK	eGFP	Gastrocnemius	Lumbar ventral horn
Nakajima *et al*.^31^	100 μl of 5.0 × 10^8 ^pfu/ml	Wistar Rats	12–14 weeks	3 days-4 weeks	CAG	LacZ	Middle section of the superficial layer of the Sternomastoid muscle	Medulla-C8
Nakajima *et al*.^32^	100 μl of 5.0 × 10^8 ^pfu/ml	SD Rats	8–10 weeks	3 days-4 weeks	CAG	BDNF, LacZ	Middle section of the superficial layer of the Sternomastoid muscle	Medulla-C8
Uchida *et al*.^33^	2.5 μl of 5 × 10^10^ pfu/ml	Twy mice	16 weeks old	1–4 weeks	CAG	NT-3, LacZ	Middle belly of the superficial layer of the Sternomastoid muscle	Medulla- C7
Nakajima *et al*.^29^	100 μl of 5.0 × 10^9^ pfu/ml	SD Rats	8–10 weeks	1–6 weeks	CAG	BDNF, LacZ	Middle belly of the superficial layer of the Sternomastoid muscle	Medulla-C7
Uchida *et al*.^25^	25 μl of 1.0 × 10^8^ pfu/ml	Twy mice	18 week old	4 weeks	CAG	BDNF, LacZ	Middle belly of the superficial layer of the Sternomastoid muscle	Medulla- C3

This table highlights the diversity in adenoviral-mediated gene delivery methods utilised in the last 20 years. β-Gal: beta-galactosidase; BDNF: brain-derived neurotrophic factor; CAG: CMV-enhancer chicken β–actin hybrid promoter; CMV: cytomegalovirus immediately early promoter; CNTF: cilliary-derived neurotrophic factor; GDNF: glial-derived neurotrophic factor; Luc: luciferase; LacZ: lactose operon Z; NGF: nerve growth factor; NT-3: neurotrophin-3; RSV: Rous-sarcoma virus long terminal repeat promoter.

## References

[b1] Liu Y., Kim D., Himes B. T., Chow S. Y., Schallert T., Murray M. . Transplants of fibroblasts genetically modified to express BDNF promote regeneration of adult rat rubrospinal axons and recovery of forelimb function. Journal of Neuroscience 19(11), 4370–4387 (1999). 10341240 10.1523/JNEUROSCI.19-11-04370.1999 PMC6782629

[b2] Ruitenberg M. J., Plant G. W., Hamers F. P. T., Wortel J., Blits B., Dijkhuizen P. A. . *Ex vivo* adenoviral vector-mediated neurotrophin gene transfer to olfactory ensheathing glia: effects on rubrospinal tract regeneration, lesion size, and functional recovery after implantation in the injured rat spinal cord. Journal of Neuroscience, 23(18), 7045–7058 (2003). 12904465 10.1523/JNEUROSCI.23-18-07045.2003 PMC6740651

[b3] Taylor L., Jones L., Tuszynski M. H. & Blesch A. Neurotrophin-3 gradients established by lentiviral gene delivery promote short-distance axonal bridging beyond cellular grafts in the injured spinal cord. Journal of Neuroscience 26(38), 9713–9721 (2006). 16988042 10.1523/JNEUROSCI.0734-06.2006 PMC6674461

[b4] Gransee H. M., Zhan W.-Z., Sieck G. C. & Mantilla C. B. Localized Delivery of Brain-Derived Neurotrophic Factor-Expressing Mesenchymal Stem Cells Enhances Functional Recovery following Cervical Spinal Cord Injury. Journal of Neurotrauma (2014). 10.1089/neu.2014.3464 PMC4298751 25093762

[b5] Fortun J., Puzis R., Pearse D. D., Gage F. H. & Bunge M. B. Muscle injection of AAV-NT3 promotes anatomical reorganization of CST axons and improves behavioral outcome following SCI. Journal of Neurotrauma 26(7), 941–953 (2009). 19275471 10.1089/neu.2008.0807

[b6] Lu P., Blesch A., Graham L., Wang Y., Samara R., Banos K. . Motor axonal regeneration after partial and complete spinal cord transection. Journal of Neuroscience 32(24), 8208–8218 (2012). 22699902 10.1523/JNEUROSCI.0308-12.2012 PMC3407545

[b7] Fouad K., Bennett D. J., Vavrek R. & Blesch A. Long-term viral brain-derived neurotrophic factor delivery promotes spasticity in rats with a cervical spinal cord hemisection. Frontiers in Neurology 4, 187 (2013). 24312075 10.3389/fneur.2013.00187 PMC3832889

[b8] Ziemlińska E., Kügler S., Schachner M., Wewiór I., Czarkowska-Bauch J. & Skup M. Overexpression of BDNF Increases Excitability of the Lumbar Spinal Network and Leads to Robust Early Locomotor Recovery in Completely Spinalized Rats. PLoS ONE 9(2), e88833 (2014). 24551172 10.1371/journal.pone.0088833 PMC3925164

[b9] Blesch A. & Tuszynski M. H. Transient Growth Factor Delivery Sustains Regenerated Axons after Spinal Cord Injury. Journal of Neuroscience 27(39), 10535–10545 (2007). 17898225 10.1523/JNEUROSCI.1903-07.2007 PMC6673161

[b10] Zhang Y., Dijkhuizen P. A., Anderson P. N., Lieberman A. R. & Verhaagen J. NT-3 delivered by an adenoviral vector induces injured dorsal root axons to regenerate into the spinal cord of adult rats. Journal of Neuroscience Research 54(4), 554–562 (1998). 9822165 10.1002/(SICI)1097-4547(19981115)54:4<554::AID-JNR12>3.0.CO;2-M

[b11] Koda M., Hashimoto M., Murakami M., Yoshinaga K., Ikeda O., Yamazaki M. . Adenovirus vector-mediated *in vivo* gene transfer of brain-derived neurotrophic factor (BDNF) promotes rubrospinal axonal regeneration and functional recovery after complete transection of the adult rat spinal cord. Journal of Neurotrauma 21(3), 329–337 (2004). 15115607 10.1089/089771504322972112

[b12] Smith G. M. & Romero M. I. Adenoviral-mediated gene transfer to enhance neuronal survival, growth, and regeneration. Journal of Neuroscience Research 55(2), 147–157 (1999). 9972817 10.1002/(SICI)1097-4547(19990115)55:2<147::AID-JNR2>3.0.CO;2-8

[b13] Harvey B.-G., Maroni J., O’Donoghue K. A., Chu K. W., Muscat J. C., Pippo A. L. . Safety of Local Delivery of Low- and Intermediate-Dose Adenovirus Gene Transfer Vectors to Individuals with a Spectrum of Morbid Conditions. Human Gene Therapy 13(1), 15–63 (2004). 11779412 10.1089/10430340152712638

[b14] Yang Z.-J., Zhang Y.-R., Chen B., Zhang S.-L., Jia E.-Z., Wang L.-S. . Phase I clinical trial on intracoronary administration of Ad-hHGF treating severe coronary artery disease. Molecular Biology Reports 36(6), 1323–1329 (2008). 18649012 10.1007/s11033-008-9315-3

[b15] Crystal R. G. Adenovirus: The First Effective *In Vivo* Gene Delivery Vector. Human Gene Therapy, 25(1), 3–11 (2014). 24444179 10.1089/hum.2013.2527 PMC3900005

[b16] Morris R. Neurotoxicity and Neuroprotection in Spinal Cord Injury. In Handbook of Neurotoxicity (pp. 1457–1482). New York, NY: Springer: New York, (2014).

[b17] Ding X., Cai J., Li S., Liu X. D., Wan Y. & Xing G. G. BDNF contributes to the development of neuropathic pain by induction of spinal long-term potentiation via SHP2 associated GluN2B-containing NMDA receptors activation in rats with spinal nerve ligation. Neurobiol Disease 73, 428–451 (2015). 10.1016/j.nbd.2014.10.025 25447233

[b18] Eleftheriadou I., Trabalza A., Ellison S. M., Gharun K. & Mazarakis N. D. Specific Retrograde Transduction of Spinal Motor Neurons Using Lentiviral Vectors Targeted to Presynaptic NMJ Receptors. Molecular Therapy: the Journal of the American Society of Gene Therapy (2014). 10.1038/mt.2014.49 PMC4089010 24670531

[b19] Kaspar B. K., Lládo J., Sherkat N., Rothstein J. D. & Gage F. H. Retrograde viral delivery of IGF-1 prolongs survival in a mouse ALS model. Science 301(5634), 839–842 (2003). 12907804 10.1126/science.1086137

[b20] Allodi I., Comley L., Nichterwitz S., Nizzardo M., Simone C., Benitez J. A. . Differential neuronal vulnerability identifies IGF-2 as a protective factor in ALS. Scientific Reports 6, 25960 (2016). 27180807 10.1038/srep25960 PMC4867585

[b21] Ghadge G. D., Roos R. P., Kang U. J., Wollmann R., Fishman P. S., Kalynych A. M. . CNS gene delivery by retrograde transport of recombinant replication-defective adenoviruses. Gene Therapy 2(2), 132–137 (1995). 7536617

[b22] Baumgartner B. J. & Shine H. D. Targeted transduction of CNS neurons with adenoviral vectors carrying neurotrophic factor genes confers neuroprotection that exceeds the transduced population. The Journal of Neuroscience: the Official Journal of the Society for Neuroscience 17(17), 6504–6511 (1997). 9254662 10.1523/JNEUROSCI.17-17-06504.1997 PMC6573127

[b23] Haase G., Kennel P., Pettmann B., Vigne E., Akli S., Revah F. . Gene therapy of murine motor neuron disease using adenoviral vectors for neurotrophic factors. Nature Medicine 3(4), 429–436 (1997). 10.1038/nm0497-429 9095177

[b24] Glatzel M., Flechsig E., Navarro B., Klein M. A., Paterna J. C., Büeler H. & Aguzzi A. Adenoviral and adeno-associated viral transfer of genes to the peripheral nervous system. Proceedings of the National Academy of Sciences of the United States of America, 97(1), 442–447 (2000). 10618437 10.1073/pnas.97.1.442 PMC26682

[b25] Uchida K., Nakajima H., Hirai T., Yayama T., Chen K., Guerrero A. R. . The retrograde delivery of adenovirus vector carrying the gene for brain-derived neurotrophic factor protects neurons and oligodendrocytes from apoptosis in the chronically compressed spinal cord of twy/twy mice. Spine 37(26), 2125–2135 (2012). 22648027 10.1097/BRS.0b013e3182600ef7

[b26] Baumgartner B. J. & Shine H. D. Neuroprotection of spinal motoneurons following targeted transduction with an adenoviral vector carrying the gene for glial cell line-derived neurotrophic factor. Experimental Neurology 153(1), 102–112 (1998a). 9743571 10.1006/exnr.1998.6878

[b27] Baumgartner B. J. & Shine H. D. Permanent rescue of lesioned neonatal motoneurons and enhanced axonal regeneration by adenovirus-mediated expression of glial cell-line-derived neurotrophic factor. Journal of Neuroscience Research 54(6), 766–777 (1998b). 9856860 10.1002/(SICI)1097-4547(19981215)54:6<766::AID-JNR4>3.0.CO;2-A

[b28] Martinov V. N., Sefland I., Walaas S. I., Lømo T., Njå A. & Hoover F. Targeting functional subtypes of spinal motoneurons and skeletal muscle fibres *in vivo* by intramuscular injection of adenoviral and adeno-associated viral vectors. Anatomy and Embryology 205(3), 215–221 (2002). 12107491 10.1007/s00429-002-0233-1

[b29] Nakajima H., Uchida K., Yayama T., Kobayashi S., Guerrero A. R., Furukawa S. & Baba H. Targeted retrograde gene delivery of brain-derived neurotrophic factor suppresses apoptosis of neurons and oligodendroglia after spinal cord injury in rats. Spine 35(5), 497–504 (2010). 20190624 10.1097/BRS.0b013e3181b8e89b

[b30] Tosolini A. P. & Morris R. Viral-mediated gene therapy for spinal cord injury (SCI) from a translational neuroanatomical perspective. Neural Regen Res 11(5), 743–744 (2016). 27335556 10.4103/1673-5374.182698 PMC4904463

[b31] Nakajima H., Uchida K., Kobayashi S., Kokubo Y., Yayama T., Sato R. & Baba H. Targeted retrograde gene delivery into the injured cervical spinal cord using recombinant adenovirus vector. Neuroscience Letters 385(1), 30–35 (2005). 15936879 10.1016/j.neulet.2005.05.012

[b32] Nakajima H., Uchida K., Kobayashi S., Inukai T., Horiuchi Y., Yayama T. . Rescue of Rat Anterior Horn Neurons after Spinal Cord Injury by Retrograde Transfection of Adenovirus Vector Carrying Brain-Derived Neurotrophic Factor Gene. Journal of Neurotrauma 24(4), 703–712 (2007). 17439352 10.1089/neu.2006.0004

[b33] Uchida K., Nakajima H., Inukai T., Takamura T., Kobayashi S., Furukawa S. & Baba H. Adenovirus-mediated retrograde transfer of neurotrophin-3 gene enhances survival of anterior horn neurons oftwy/twy mice with chronic mechanical compression of the spinal cord. Journal of Neuroscience Research 86(8), 1789–1800 (2008). 18253945 10.1002/jnr.21627

[b34] Tosolini A. P. & Morris R. Spatial characterization of the motor neuron columns supplying the rat forelimb. Neuroscience, 200, 19–30 (2012). 22100785 10.1016/j.neuroscience.2011.10.054

[b35] Tosolini A. P., Mohan R. & Morris R. Targeting the Full Length of the Motor End Plate Regions in the Mouse Forelimb Increases the Uptake of Fluoro-Gold into Corresponding Spinal Cord Motor Neurons. Frontiers in Neurology 4, 1–10 (2013). 23730296 10.3389/fneur.2013.00058 PMC3657688

[b36] Mohan R., Tosolini A. P. & Morris R. Targeting the motor end plates in the mouse hindlimb gives access to a greater number of spinal cord motor neurons: An approach to maximize retrograde transport. Neuroscience 274, 318–330 (2014). 24892760 10.1016/j.neuroscience.2014.05.045

[b37] Mohan R., Tosolini A. P. & Morris R. Intramuscular Injections Along the Motor End Plates: a Minimally Invasive Approach to Shuttle Tracers Directly into Motor Neurons. J. Vis. Exp. e52846 (2015a) 26273739 10.3791/52846 PMC4545031

[b38] Mohan R., Tosolini A. P. & Morris R. Segmental distribution of the motor neuron columns that supply the rat hindlimb: A muscle/motor neuron tract-tracing analysis targeting the motor end plates. Neuroscience, 307, 98–108 (2015). 26304758 10.1016/j.neuroscience.2015.08.030

[b39] Kelkar S. A., Pfister K. K., Crystal R. G. & Leopold P. L. Cytoplasmic dynein mediates adenovirus binding to microtubules. Journal of Virology 78(18), 10122–10132 (2004). 15331745 10.1128/JVI.78.18.10122-10132.2004 PMC515014

[b40] Sinnreich M., Shaw C. A., Pari G., Nalbantoglu J., Holland P. C. & Karpati G. Localization of coxsackie virus and adenovirus receptor (CAR) in normal and regenerating human muscle. Neuromuscular Disorders 15(8), 541–548 (2005). 16014330 10.1016/j.nmd.2005.05.007

[b41] Arnberg N. Adenovirus receptors: implications for targeting of viral vectors. Trends in Pharmacological Sciences 33(8), 442–448 (2012). 22621975 10.1016/j.tips.2012.04.005

[b42] Shaw C. A., Holland P. C., Sinnreich M., Allen C., Sollerbrant K., Karpati G. & Nalbantoglu J. Isoform-specific expression of the Coxsackie and adenovirus receptor (CAR) in neuromuscular junction and cardiac intercalated discs. BMC Cell Biology, 5(1), 42 (2004). 15533241 10.1186/1471-2121-5-42 PMC533869

[b43] Boulis N. M., Bhatia V., Brindle T. I., Holman H. T., Krauss D. J., Blaivas M. & Hoff J. T. Adenoviral nerve growth factor and beta-galactosidase transfer to spinal cord: a behavioral and histological analysis. Journal of Neurosurgery 90(1), 99–108 (1999). 10413133 10.3171/spi.1999.90.1.0099

[b44] Huber A. B., Ehrengruber M. U., Schwab M. E. & Brösamle C. Adenoviral gene transfer to the injured spinal cord of the adult rat. The European Journal of Neuroscience 12(9), 3437–3442 (2000). 10998127 10.1046/j.1460-9568.2000.00255.x

[b45] Yamashita S., Mita S., Arima T., Maeda Y., Kimura E., Nishida Y. . Bcl-2 expression by retrograde transport of adenoviral vectors with Cre-loxP recombination system in motor neurons of mutant SOD1 transgenic mice. Gene Therapy 8(13), 977–986 (2001). 11438832 10.1038/sj.gt.3301479

[b46] Acsadi G., Anguelov R. A., Yang H., Toth G., Thomas R., Jani A. . Increased Survival and Function of SOD1 Mice After Glial Cell-Derived Neurotrophic Factor Gene Therapy. Human Gene Therapy, 13(9), 1047–1059 (2002). 12067438 10.1089/104303402753812458

[b47] Kraut R., Menon K. & Zinn K. A gain-of-function screen for genes controlling motor axon guidance and synaptogenesis in Drosophila. Current Biology, 11(6), 417–430 (2001). 11301252 10.1016/s0960-9822(01)00124-5

[b48] Kratsios P., Pinan-Lucarré B., Kerk S. Y., Weinreb A., Bessereau J.-L. & Hobert O. Transcriptional Coordination of Synaptogenesis and Neurotransmitter Signaling. Current Biology 25(10), 1282–1295 (2015). 25913400 10.1016/j.cub.2015.03.028 PMC4465358

[b49] Alto L. T., Havton L. A., Conner J. M., Hollis E. R. II, Blesch A. & Tuszynski M. H. Chemotropic guidance facilitates axonal regeneration and synapse formation after spinal cord injury. Nature Publishing Group 12(9), 1106–1113 (2009). 10.1038/nn.2365 PMC2753201 19648914

[b50] Steward O., Zheng B. & Tessier-Lavigne M. False resurrections: distinguishing regenerated from spared axons in the injured central nervous system. The Journal of Comparative Neurology, 459(1), 1–8 (2003). 12629662 10.1002/cne.10593

[b51] Hordeaux J., Dubreil L., Deniaud J., Iacobelli F., Moreau S., Ledevin M. . Efficient central nervous system AAVrh10-mediated intrathecal gene transfer in adult and neonate rats. Gene Therapy 22(4), 316–324 (2015). 25588740 10.1038/gt.2014.121

[b52] Von Jonquieres G., Mersmann N., Klugmann C. B., Harasta A. E., Lutz B., Teahan O. . Glial Promoter Selectivity following AAV-Delivery to the Immature Brain. PLoS ONE 8(6), e65646 (2013). 23799030 10.1371/journal.pone.0065646 PMC3683058

[b53] Nalbantoglu J., Pari G., Karpati G. & Holland P. C. Expression of the primary coxsackie and adenovirus receptor is downregulated during skeletal muscle maturation and limits the efficacy of adenovirus-mediated gene delivery to muscle cells. Human Gene Therapy 10(6), 1009–1019 (1999). 10223734 10.1089/10430349950018409

[b54] Acsadi G., Jani A., Massie B., Simoneau M., Holland P., Blaschuk K. & Karpati G. A differential efficiency of adenovirus-mediated *in vivo* gene transfer into skeletal muscle cells of different maturity. Human Molecular Genetics 3(4), 579–584 (1994). 8069302 10.1093/hmg/3.4.579

[b55] Nalbantoglu J., Larochelle N., Wolf E., Karpati G., Lochmüller H. & Holland P. C. Muscle-specific overexpression of the adenovirus primary receptor CAR overcomes low efficiency of gene transfer to mature skeletal muscle. Journal of Virology 75(9), 4276–4282 (2001). 11287577 10.1128/JVI.75.9.4276-4282.2001 PMC114173

[b56] Larochelle N., Teng Q., Gilbert R., Deol J. R., Karpati G., Holland P. C. & Nalbantoglu J. Modulation of coxsackie and adenovirus receptor expression for gene transfer to normal and dystrophic skeletal muscle. The Journal of Gene Medicine, 12(3), 266–275 (2010). 20082422 10.1002/jgm.1433

[b57] Darabid H., Perez-Gonzalez A. P. & Robitaille R. Neuromuscular synaptogenesis: coordinating partners with multiple functions. Nature Reviews Neuroscience 15(11), 703–718 (2014). 25493308

[b58] Sleigh J. N., Grice S. J., Burgess R. W., Talbot K. & Cader M. Z. Neuromuscular junction maturation defects precede impaired lower motor neuron connectivity in Charcot–Marie–Tooth type 2D mice. Human Molecular Genetics 23(10), 2639–2650 (2014). 24368416 10.1093/hmg/ddt659 PMC3990164

[b59] Sleigh J. N. & Schiavo G. Older but not slower: aging does not alter axonal transport dynamics of signalling endosomes *in vivo*. Matters. 10.19185/matters.201605000018 (2016).

[b60] Zirger J. M., Puntel M., Bergeron J., Wibowo M., Moridzadeh R., Bondale N. . Immune-mediated Loss of Transgene Expression From Virally Transduced Brain Cells Is Irreversible, Mediated by IFNγ, Perforin, and TNFα, and due to the Elimination of Transduced Cells. Molecular Therapy: the Journal of the American Society of Gene Therapy 20(4), 808–819 (2012). 22233583 10.1038/mt.2011.243 PMC3321600

[b61] Lowenstein P. R., Mandel R. J., Xiong W.-D., Kroeger K. & Castro M. G. Immune responses to adenovirus and adeno-associated vectors used for gene therapy of brain diseases: the role of immunological synapses in understanding the cell biology of neuroimmune interactions. Current Gene Therapy 7(5), 347–360 (2007). 17979681 10.2174/156652307782151498 PMC2268649

[b62] Petrof B. J., Acsadi G., Jani A., Massie B., Bourdon J., Matusiewicz N. . Efficiency and functional consequences of adenovirus-mediated *in vivo* gene transfer to normal and dystrophic (mdx) mouse diaphragm. American Journal of Respiratory Cell and Molecular Biology, 13(5), 508–517 (1995). 7576685 10.1165/ajrcmb.13.5.7576685

[b63] Boulis N. M., Turner D. E., Imperiale M. J. & Feldman E. L. Neuronal survival following remote adenovirus gene delivery. Journal of Neurosurgery 96(2) (Suppl), 212–219 (2002). 12450285 10.3171/spi.2002.96.2.0212

[b64] Chen Y., Müller J. D., Ruan Q. & Gratton E. Molecular brightness characterization of EGFP *in vivo* by fluorescence fluctuation spectroscopy. Biophysical Journal, 82(1) (Pt 1), 133–144 (2002). 11751302 10.1016/S0006-3495(02)75380-0 PMC1302455

[b65] Morris R., Tosolini A. P., Goldstein J. D. & Whishaw I. Q. Impaired Arpeggio Movement in Skilled Reaching by Rubrospinal Tract Lesions in the Rat: A Behavioral/Anatomical Fractionation. Journal of Neurotrauma 28(12), 2439–2451 (2011). 21612320 10.1089/neu.2010.1708

[b66] Watson C. & Harrison M. The Location of the Major Ascending and Descending Spinal Cord Tracts in all Spinal Cord Segments in the Mouse: Actual and Extrapolated. Anatomical Record (Hoboken N.J., 2007), 295(10), 1692–1697 (2012). 10.1002/ar.22549 22847889

[b67] Kerman I. A., Enquist L. W., Watson S. J. & Yates B. J. Brainstem substrates of sympatho-motor circuitry identified using trans-synaptic tracing with pseudorabies virus recombinants. Journal of Neuroscience 23(11), 4657–4666 (2003). 12805305 10.1523/JNEUROSCI.23-11-04657.2003 PMC6740797

[b68] Kurz E. M., Sengelaub D. R. & Arnold A. P. Androgens regulate the dendritic length of mammalian motoneurons in adulthood. Science 232(4748), 395–398 (1986). 3961488 10.1126/science.3961488

[b69] Giménez y Ribotta M., Revah F., Pradier L., Loquet I., Mallet J. & Privat A. Prevention of motoneuron death by adenovirus-mediated neurotrophic factors. Journal of Neuroscience Research 48(3), 281–285 (1997). 9160251 10.1002/(sici)1097-4547(19970501)48:3<281::aid-jnr11>3.3.co;2-i

[b70] Soudais C., Laplace-Builhe C., Kissa K. & Kremer E. J. Preferential transduction of neurons by canine adenovirus vectors and their efficient retrograde transport *in vivo*. FASEB Journal: Official Publication of the Federation of American Societies for Experimental Biology 15(12), 2283–2285 (2001). 11511531 10.1096/fj.01-0321fje

[b71] Millecamps S., Mallet J. & Barkats M. Adenoviral retrograde gene transfer in motoneurons is greatly enhanced by prior intramuscular inoculation with botulinum toxin. Human Gene Therapy 13(2), pp. 225–232 (2002). 11812279 10.1089/10430340252769752

[b72] Yamashita S., Mita S., Kato S., Okado H., Ohama E. & Uchino M. Bcl-2 expression using retrograde transport of adenoviral vectors inhibits cytochrome c-release and caspase-1 activation in motor neurons of mutant superoxide dismutase 1 (G93A) transgenic mice. Neuroscience Letters 350(1), 17–20 (2003). 12962907 10.1016/s0304-3940(03)00817-6

[b73] Tsai L.-K., Tsai M.-S., Shyue S.-K., Hwu W.-L. & Li H. Adenoviral interneuronal transportation after retrograde gene transfer in mice. Molecular Brain Research 142(2), 151–155 (2005). 16297496 10.1016/j.molbrainres.2005.09.021

[b74] Haase G., Pettmann B., Vigne E., Castelnau-Ptakhine L. Schmalbruch H. & Kahn A. Adenovirus-mediated transfer of the neurotrophin-3 gene into skeletal muscle of pmn mice: Therapeutic effects and mechanisms of action. Journal of the Neurological Sciences 160, S97–S105 (1998). 9851658 10.1016/s0022-510x(98)00207-x

